# 5-allyl-7-gen-difluoromethoxychrysin enhances TRAIL-induced apoptosis in human lung carcinoma A549 cells

**DOI:** 10.1186/1471-2407-11-322

**Published:** 2011-07-29

**Authors:** Zhao-Hui Xie, Mei-Fang Quan, Fei Liu, Jian-Guo Cao, Jian-Song Zhang

**Affiliations:** 1 Changsha Health Vocational College, Changsha 410010, Hunan Province, China; 2 Medical College, Hunan Normal University, Changsha 410013, Hunan Province, China

**Keywords:** Lung cancer, chrysin, 5-allyl-7-gen-difluoromethoxychrysin, TRAIL, apoptosis, therapeutic action

## Abstract

**Background:**

5-allyl-7-gen-difluoromethoxychrysin (AFMC) is a novel synthetic analogue of chrysin that has been reported to inhibit proliferation in various cancer cell lines. Tumor necrosis factor-related apoptosis-inducing ligand (TRAIL) is a promising anti-cancer agent.

**Methods:**

The cytotoxicity of A549 and WI-38 cells were determined using colorimetry. Apoptosis was detected by flow cytometry (FCM) after propidium iodide (PI) fluorescence staining and agarose gel electrophoresis. Caspase activities were evaluated using enzyme-linked immunosorbent assay (ELISA).The expressions of DR4 and DR5 were analyzed using FCM and western blot.

**Results:**

Subtoxic concentrations of AFMC sensitize human non-small cell lung cancer (NSCLC) A549 cells to TRAIL-mediated apoptosis. Combined treatment of A549 cells with AFMC and TRAIL significantly activated caspase-3, -8 and -9. The caspase-3 inhibitor zDEVD-fmk and the caspase-8 inhibitor zIETD-fmk blocked the apoptosis of A549 cells induced by co-treatment with AFMC and TRAIL. In addition, we found that treatment of A549 cells with AFMC significantly induced the expression of death receptor 5 (DR5). AFMC-mediated sensitization of A549 cells to TRAIL was efficiently reduced by administration of a blocking antibody or small interfering RNAs against DR5. AFMC also caused increase of the Sub-G1 cells by TRAIL treatment and increased the expression levels of DR5 in other NSCLC H460 and H157 cell lines. In contrast, AFMC-mediated induction of DR5 expression was not observed in human embryo lung WI-38 cells, and AFMC did not sensitize WI-38 cells to TRAIL-induced apoptosis.

**Conclusions:**

AFMC synergistically enhances TRAIL-mediated apoptosis in NSCLC cells through up-regulating DR5 expression.

## Background

Lung cancer is the leading cause of cancer deaths in the world with over one million cases diagnosed every year. Multiple options for the treatment of lung cancer have been described, including surgery, chemotherapy and radiation; however, therapeutic efficacy is typically transient and mostly absent with advanced disease [1,2]. Therefore, the need for more rational approaches to lung cancer therapy is essential.

Chrysin (5, 7-dihydroxyflavone) is a naturally occurring flavonoid possessing a broad range of pharmacological activities and is widely found in fruits, vegetables, honey and propolis [3,4]. Recent studies have shown that chrysin in diets is a promising biological anti-cancer agent. Chrysin has been demonstrated to induce apoptosis of human myeloid leukemia cells via activation of caspases and inactivation of Akt [5]. Our previous studies demonstrated that chrysin and its derivatives exhibited significant anticancer effect against gastric cancer SGC-7901 cells and colorectal cancer HT-29 cells [6,7]. However, administration of chrysin alone is insufficient. The combination of chrysin with other anti-cancer agents or modifications to its structure may improve the biological activity of chrysin.

Our previous studies showed that inhibition of proliferation and induction of apoptosis by 5,7-dihydrox-8-nitrochrysin and 8-bromo-7-methoxychrysin in human gastric carcinoma SGC-7901 cells and hepatocellular carcinoma cells, respectively, was stronger than that of the lead compound chrysin [8,9]. 5-allyl-7-gen-difluoromethoxychrysin (AFMC) is another important derivative of chrysin. We previously reported that AFMC inhibited the proliferation of the ovarian cancer CoC1 cell line and hepatocellular carcinoma HepG2 cell line [10,11]. However, whether AFMC possesses antitumor effects on human non-small cell lung cancer (NSCLC) cells and the molecular mechanisms of its action remain to be determined.

Tumor necrosis factor-related apoptosis-inducing ligand (TRAIL) is a member of the tumor necrosis factor (TNF) superfamily, which includes potent inducers of apoptosis in a wide variety of cancer cells [12-14]. In particular, TRAIL is considered as the most promising anticancer agent in the TNF superfamily because of its selective cytotoxicity against tumor cells versus normal primary cells. TRAIL induces apoptosis in various cancer cells through its interaction with death receptor 5 (DR5), which contains a cytoplasmic death domain capable of recruiting apoptosis signaling molecules and inducing apoptosis [15-21]. However, many tumor cells are resistant to TRAIL-induced apoptosis [22,23]. Various cancer therapeutic agents have been demonstrated to augment TRAIL-induced apoptosis through induction of DR5 expression [24-30], indicating that DR5 expression levels might be involved in resistance to TRAIL. Thus, induction of DR5 expression could enhance cytotoxicity and apoptosis mediated by TRAIL.

In this study, we investigated the apoptotic effects of AFMC in combination with TRAIL on NSCLC cells. We show for the first time that AFMC synergistically enhances TRAIL-mediated apoptosis in NSCLC cells through up-regulating DR5 expression. Thus, our findings raise the possibility that combined use of AFMC and TRAIL could be a candidate therapy for the treatment of human NSCLC.

## Methods

### Cell lines and cell culture

Human non-small-cell lung carcinoma cell lines A549, which possess wild-type p53 [31], were purchased from China Center for Type Culture Collection (CCTCC, Wuhan, China). Human lung cancer cell lines H460, which possess wild-type p53 [31], H157, which express mutant p53 [31], and human embryo lung WI-38 cells were purchased from Cell Bank, Chinese Academy of Sciences (Shanghai, China). A549 and WI-38 cells were cultured in RPMI-1640 medium with 10% fetal bovine serum, 100 units/mL penicillin and 100 μg/mL streptomycin (Life Technologies, Inc) at 37°C in a 5% CO_2 _incubator. H460 and H157 cells were maintained in DMEM supplemented with 10% bovine calf serum, 1 mmol/L sodium pyruvate, 2 mmol/L L-glutamine and 50 μg/mL penicillin/streptomycin.

### Reagents

AFMC was synthesized as previously described [6]. AFMC was dissolved in dimethyl sulfoxide (DMSO), diluted with phosphate buffer solution (PBS), and prepared at 2 mmol/L in a storage solution after filtration sterilization. Chrysin was purchased from Sigma (St. Louis, MO). RPMI-1640 was purchased from Gibco Company. Fetal bovine serum was from Hangzhou Sijiqing Biological Engineering Materials Co., Ltd. Trypsin and DMSO was from Amersco Company. The Apoptotic DNA Ladder Detection Kit was purchased from Bodataike Company (Beijing, China). Caspase 3 Activity Detection Kit, Caspase 8 Colorimetric Activity Assay Kit 25, and Caspase 9 Colorimetric Activity Assay Kit were from Millipore, (Billerica, MA, U.S.A.). Mouse anti-human DR5 and DR4 monoclonal antibodies were from Santa Cruz Biotechnology, Inc (Santa Cruz, CA, U.S.A). Fluorescein isothiocyanate (FITC)-conjugated anti-mouse IgG was from Zymed Laboratories(South San Francisco, CA, USA). Mouse IgG1 immunoglobulin was from DakoCytomation(Glostrup, Denmark). Recombinant human TRAIL was purchased from PeproTech, Inc. (London, United Kingdom). Lipofectamine 2000 reagent was from Invitrogen Company. DR5/Fc chimera protein and caspase inhibitors (zDEVD-fmk for caspase-3, zIETD-fmk for caspase-8 and zLEHD-fmk for caspase-9) were from R&D Systems (Minneapolis, MN).

### Lactate dehydrogenase (LDH) release assay

Cells were seeded in 24-well plates at a density of 1.0 × 10^4 ^cells/well as previously described [30]. Drugs of different concentrations were added to each well and cultured for 48 h. Supernatants (500 μL) were then removed from each sample for measurement of LDH release. The absorbance of the sample was read at 490 nm using an automatic biochemistry analyzer. All treatments and control samples were analyzed in triplicate. Cytotoxicity (%) was calculated as (*A*_490 _of treated group)/( *A*_490 _of positive control group), where the positive control was the absorbance of cells cultured in medium containing 100 mmol/L H_2_O_2_.

### Detection of apoptosis and caspase activity

Apoptosis was detected by flow cytometry (FCM) after propidium iodide (PI) fluorescence staining as described previously [32]. DNA ladder bands were determined by agarose gel electrophoresis. Caspase activity assays were evaluated using enzyme-linked immunosorbent assay (ELISA) as previously described [33]. Fold increases in caspase activity were calculated as (A_405 _of treated group)/(A_405 _of negative control group), where the negative control was the absorbance of cells cultured in medium containing only 0.2% DMSO. In the caspase inhibition assays, cells were pretreated with a caspase-specific inhibitor (10 μmol/L zDEVD-fmk, zIETD-fmk, or zLEHD-fmk) for 1 h prior to addition of agents tested.

### Analysis of cell surface receptor expression

Cells were cultured as indicated concentration and for 24 h, and then collected. Five hundred thousand cells for each receptor analysis were transferred to polystyrene tubes, washed twice with PBS and resuspended in PBS containing 0.5% BSA (Sigma). A specific monoclonal antibody to either DR5, DR4 or unspecific mouse IgG1 as isotype control was applied at 5 μg/mL. Cells were incubated for 20 min with gentle rocking at the room temperature. Cells were washed twice in PBS and secondary fluorescein isothiocyanate-conjugated polyclonal goat antibody to mouse IgG1 (1:200 in PBS containing 0.5% BSA) was added, followed by incubation protected from light for 30 min with gentle rocking at the room temperature. Cells were then washed and resuspended in PBS containing 0.5% BSA. All analysis was carried out on an EPICS XL flow cytometer (Coulter, Miami, FL) using CellQuest software (Pharmingen BD Biosciences).

### Silencing of DR5 expression using small interfering RNA (siRNA)

Silencing of DR5 expression in A549 cells was performed as previously described by Wang *et al*. [34]. High-purity control (LacZ) and DR5 siRNA oligos were synthesized by Shanghai Sangon Biotech Co., Ltd and consisted of 21 base pairs. The sequences are as follows:DR5, 5'-AAGACCCUUGUGCUCGUUGUC-3', and LacZ, 5'-AACGUACGCGGAAUACUUCGA-3'. siRNA transfection was performed using the Lipofectamine 2000 reagent following the manufacturer's instructions. Forty-eight hours after transfection, cells were treated with TRAIL and AFMC alone or in combination for another 24 h. Gene-silencing efficiency was evaluated by Western blotting using mouse anti-human DR5 monoclonal antibody. Apoptotic rate was measured by FCM after PI staining and caspase activation was determined using ELISA.

### Western blot analysis

The procedures for preparation of whole cell lysates and Western blot analysis have been previously described [35]. Anti-DR5, anti-DR4 and anti-β-actin antibodies were used as primary antibodies. Signals were detected using an ECL Advanced Western blot analysis system (Amersham Pharmacia Biotech Inc., Piscataway, NJ, USA).

### Statistical analysis

Experimental data in each group were presented as mean ± SD. Analysis of variance was performed with SPSS 15.0 software for Windows using one-way ANOVA and pair-wise comparison with *t *test. P < 0.05 was considered statistically significant.

## Results

### Subtoxic concentrations of AFMC sensitize A549 cells to TRAIL-induced apoptosis

**Figure 1:**
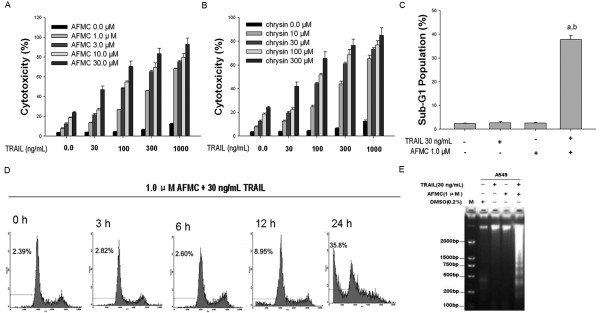
**Cytotoxic and apoptotic activities of AFMC or TRAIL or both on A549 cells**. Figure 1A: A549 cells were treated with AFMC for 30 min and then treated with or without TRAIL for 24 h at the indicated concentrations. Cytotoxicity was assessed by ELISA. Columns, average of three individual experiments; bars, SD. Figure 1B: A549 cells were treated with chrysin for 30 min and then treated with or without TRAIL for 24 h at the indicated concentrations. Cytotoxicity was assessed by ELISA. Columns, average of three individual experiments; bars, SD. Figure 1C: A549 cells treated with 1 μmol/L AFMC or 30 ng/mL TRAIL for 24 h or the pretreated with 1 μmol/L AFMC for 30 min followed by 30 ng/mL TRAIL for 24 h. DNA content of the cells was analyzed by flow cytometry. Columns, average of three individual experiments; bars, SD. a P < 0.05 vs 0.2% DMSO; b P < 0.05 vs 30 ng/mL TRAIL or 1.0 μmol/L AFMC alone. Figure 1D: A549 cells were pretreated with 1.0 μmol/L AFMC for 30 min and then treated with 30 ng/mL TRAIL for the indicated times. DNA content of the cells was analyzed by flow cytometry. Figure 1E: A549 cells were treated as Figure 1C described, fragmented DNA was extracted from treated cells and analyzed on a 2.0% agarose gel.

We first performed the LDH release assay to measure the cytotoxic effects of AFMC and TRAIL alone or in combination on NSCLC A549 cells compared to that of the lead compound chrysin. Treatment of A549 cells with 30 to 1000 ng/mL TRAIL for 24 h induced limited cytotoxicity (< 15%), suggesting that these cells were resistant to the cytotoxic effects of TRAIL. Slight cytotoxicity (< 20%) was also observed when cells were treated with 1.0 to 30.0 μmol/L AFMC. However, co-treatment of A549 cells with TRAIL and AFMC markedly increased the cytotoxic effects of TRAIL in a dose-dependent manner compared to treatment with TRAIL or AFMC alone (Figure 1A). As shown in Figure 1B, similar results were obtained when cells were treated with 10.0 to 300 μmol/L chrysin (which is equivalent to 10 times the concentration of AFMC). These results indicated that subtoxic concentrations of AFMC and chrysin sensitized TRAIL-resistant human NSCLC A549 cells to TRAIL-induced cytotoxicity and that AFMC was more potent than the lead compound chrysin. Thus, we focused our study on the effect and mechanism of AFMC.

We then investigated whether apoptosis was induced by the combination of AFMC and TRAIL using flow cytometric analysis to detect increases in hypodiploid cell populations. Co-treatment of A549 cells with 1.0 μmol/L AFMC and 30 ng/mL TRAIL for 24 h significantly induced accumulation of cells in the sub-G1 phase, which increased at 12 h and peaked at 24 h, whereas treatment with AFMC or TRAIL alone did not (Figure 1C and 1D). Furthermore, DNA fragmentation analysis by agarose gel electrophoresis showed a typical ladder pattern of inter-nucleosomal DNA fragments in A549 cells co-treated with 1.0 μmol/L AFMC and 30 ng/mL TRAIL, but not in cells treated with AFMC or TRAIL alone (Figure 1E). Collectively, these results suggested that AFMC stimulated TRAIL-induced apoptosis in A549 cells.

### Activation of caspase-8 is required for induction of apoptosis in A549 cells by combined treatment with AFMC and TRAIL

**Figure 2:**
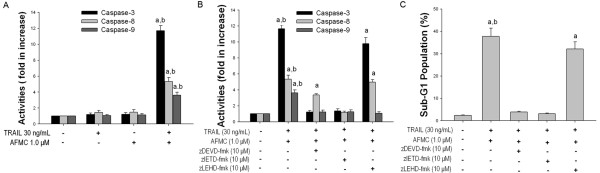
**Effects of AFMC and/or TRAIL on the caspase activities of A549 cells**. Figure 2A: Enzymatic activities of caspase -3, -8 and -9 were determined by incubation of 20 μg of total protein with 200 μmol/L chromogenic substrate (Ac-DEVD-pNA, Ac-IETD-pNA or Ac-LEHD-pNA) in 100 μL of assay buffer for 2 h at 37°C. The release of the chromophore p-nitroanilide (pNA) was monitored spectrophotometrically at 405 nm. Data are shown as mean ± S.D. (n = 3). a P < 0.05 vs. 0.2% DMSO; b P < 0.05 vs. 30 ng/mL TRAIL or 1.0 μmol/L AFMC alone. Figure 2B: A549 cells were pretreated with 1.0 μmol/L AFMC for 30 min and then treated with 30 ng/mL TRAIL for 24 h in the presence or absence of 10.0 μmol/L caspase inhibitors. Enzymatic activities of caspase -3, -8 and -9 were determined as described above. Data are shown as mean ± S.D. (n = 3). a P < 0.05 vs. 0.2% DMSO; b P < 0.05 vs. 30 ng/mL TRAIL or 1.0 μmol/L AFMC alone. Figure 2C: A549 cells were treated as described above. The frequency of cells in the sub-G1 phase was measured using flow cytometry. Data are shown as mean ± S.D. (n = 3). a P < 0.05 vs. 0.2% DMSO; b P < 0.05 vs. pretreatment with caspase inhibitors.

Monasterio *et al* [36]. reported that flavonoids, including chrysin, induced apoptosis via a mechanism that required the activation of caspase-3 and caspase-8, indicating that chrysin-induced apoptosis could act via a ligand/receptor-dependent cell death mechanism. Therefore, we next examined whether caspases were activated during the induction of apoptosis in A549 cells by the combined treatment with AFMC and TRAIL. Treatment of A549 cells with 1.0 μmol/L AFMC alone for 24 h did not induce activation of caspase-3, -8 or -9. Similarly, these caspases were not activated in response to treatment of A549 cells with 30 ng/mL TRAIL. In contrast, co-treatment with AFMC and TRAIL induced the activation of caspase-3, -8 and -9 (Figure 2A).

To further investigate caspase activation by AFMC and TRAIL co-treatment, we treated A549 cells with the caspase-3 inhibitor zDEVD-fmk, the caspase-8 inhibitor zIETD-fmk and the caspase-9 inhibitor zLEHD-fmk. As shown in Figure 2B, zDEVD-fmk and zIETD-fmk reduced the activation of caspase-3 induced by AFMC and TRAIL, whereas zLEHD-fmk had little effect. Similarly, zIETD-fmk completely blocked caspase-8 activation, and zDEVD-fmk partially blocked caspase-8, whereas zLEHD-fmk had no effect (Figure 2B). Likewise, we found that caspase-9 was activated in cells treated with AFMC plus TRAIL but not in the presence of zIETD-fmk or zLEHD-fmk (Figure 2B). Collectively, these results suggested that AFMC and TRAIL activated caspase-8 upstream of caspase-9 in A549 cells.

We further examined the role of caspase-8 activated during apoptosis using the caspase-8 inhibitor zIETD-fmk. Co-treatment with AFMC and TRAIL induced activation of caspase-3, which was disrupted by zIETD-fmk (Figure 2B). zIETD-fmk reversed the AFMC and TRAIL-induced accumulation of A549 cells in the sub-G1 phase (Figure 2C). These data indicated that apoptosis induced by AFMC and TRAIL co-treatment was dependent on caspase-8 activation.

### AFMC increases the expression of DR5 in A549 cells

**Figure 3:**
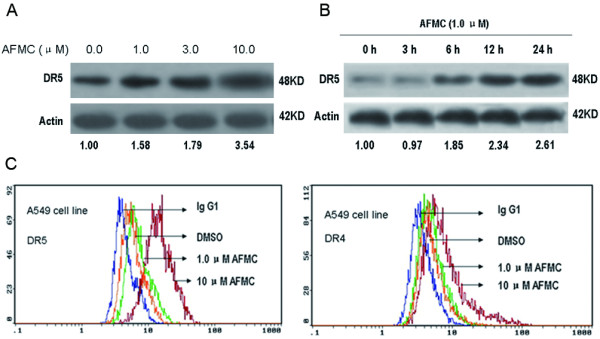
**AFMC increases DR5 protein levels in A549 cells**. Figure 3A: A549 cells were treated with the indicated concentrations of AFMC for 24 h, and DR5 levels were measured by Western blotting. β-actin was used as a loading control. Figure 3B: A549 cells were treated with 1.0 μmol/L AFMC for the indicated time, and DR5 levels were measured by Western blotting. β-actin was used as a loading control. Figure 3C: A549 cells were treated with the indicated concentrations of AFMC for 24 h, DR5 and DR4 protein levels on the surface of A549 cells were determined using flow cytometry. Mouse IgG1 was used as an isotype control.

We previously reported that AFMC induced apoptosis in the ovarian cancer CoC1 cell line and human hepatocellular carcinoma Hep G2 cell line [11,37]. TRAIL is known to interact with death receptors DR4 and DR5 to trigger apoptotic signaling pathways, and caspase-8 plays a central role in apoptosis mediated by death receptors [37-39]. To explore the molecular mechanism by which AFMC enhanced the induction of apoptosis by TRAIL in A549 cells, we first examined the effect of AFMC on the expression of DR4 and DR5 by Western blotting. As shown in Figure 3A, we found that AFMC treatmemt for 24 h significantly up-regulated DR5 expression in A549 cells in a dose-dependent manner. The increase in DR5 expression occurred as early as 6 h after treatment, which preceded the increase in hypodiploid cell populations occurring after 12 h of AFMC and TRAIL co-treatment (Figure 3B and 1D). In addition, FCM analysis showed that AFMC increased DR5 protein levels on the surface of A549 cells (Figure 3C). DR5 expression levels in A549 cells increased in a dose-dependent manner with AFMC treatment, but DR4 expression levels did not obviously change (Figure 3C).

### AFMC enhances TRAIL-induced apoptosis through upregulation of DR5 in A549 cells

**Figure 4:**
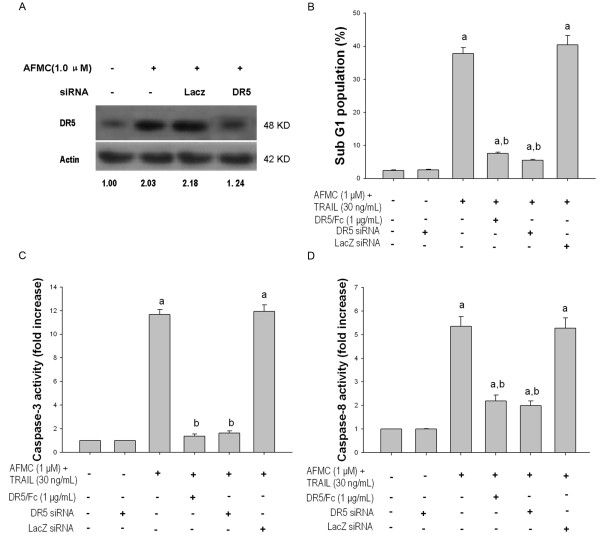
**Effects of DR5/Fc and DR5 siRNA on AFMC-induced DR5 expression and AFMC/TRAIL-induced apoptosis**. Figure 4A: A549 cells were transfected with LacZ siRNA or siRNA against DR5 followed by treatment with 1.0 μmol/L AFMC for 24 h. DR5 levels were measured by Western blotting. β-actin was used as a loading control. Figure 4B: A549 cells were preincubated with 1.0 μg/mL DR5-specific blocking chimera antibody or transfected with siRNAs, incubated for 24 h, and then treated with or without 1.0 μmol/L AFMC for 30 min followed by treatment with 30 ng/mL TRAIL for 24 h. The frequency of cells in the sub-G1 phase was measured using flow cytometry. Data are shown as mean ± S.D. (n = 3). aP < 0.05 vs. 0.2% DMSO; bP < 0.05 vs. combined treatment. Figure 4C: A549 cells were treated as described above. Enzymatic activity of caspase-3 was determined by incubation of 20 μg of total protein with 200 μM of Ac-DEVD-pNA in 100 μL of assay buffer for 2 h at 37°C. pNA release was monitored spectrophotometrically at 405 nm. Data are shown as mean ± S.D. (n = 3). aP < 0.05 vs. 0.2% DMSO; bP < 0.05 vs. combined treatment. Figure 4D: A549 cells were treated as described above. Enzymatic activity of caspase-8 was determined by incubation of 20 μg of total protein with 200 μM of Ac-IETD-pNA in 100 μL of assay buffer for 2 h at 37°C. pNA release was monitored spectrophotometrically at 405 nm. Data are shown as mean ± S.D. (n = 3). aP < 0.05 vs. 0.2% DMSO; bP < 0.05 vs. combined treatment.

To confirm that induction of apoptosis by the combination of AFMC and TRAIL occurred through DR5 upregulation, we used DR5/Fc chimera protein, a pharmacological inhibitor, and DR5 siRNA to block DR5 expression at the mRNA level, respectively. In cells transfected with DR5 siRNA, DR5 expression was reduced to basal levels (Figure 4A). FCM analysis using PI staining showed that treatment of cells with 1 μg/mL DR5/Fc or DR5 siRNA reduced the frequency of sub-G1 cells to 7.61 ± 0.32% or 5.47 ± 0.30%, respectively (Figure 4B), compared to 40.47 ± 2.73% in control siRNA-transfected cells. Collectively, these results clearly indicated that decreasing DR5 levels could efficiently block the apoptosis caused by co-treatment with AFMC and TRAIL. We also found that AFMC and TRAIL-induced caspase-8 and -3 activation was efficiently abrogated when DR5 expression was attenuated or blocked by the DR5/Fc chimera protein or DR5 siRNA transfection (Figure 4C and 4D). These results indicated that AFMC-mediated sensitization of A549 cells to TRAIL was dependent on the upregulation of DR5 expression.

### Effects of combined treatment with AFMC and TRAIL on apoptosis and DR5 expression in other NSCLC lines and in human immortalized embryo lung WI-38 cells

**Figure 5:**
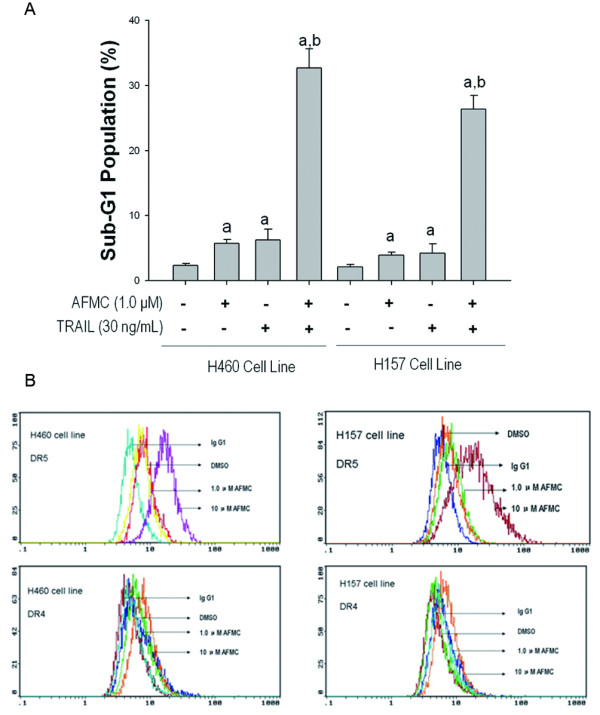
**Effects of AFMC and/or TRAIL on apoptosis and DRs expression of H460 and H157 cells**. Figure 5A: Cells were treated with 1.0 μmol/L AFMC or 30 ng/mL TRAIL for 24 h or the pretreated with 1.0 μmol/L AFMC for 30 min followed by 30 ng/mL TRAIL for 24 h. DNA content of the cells was analyzed by flow cytometry. Columns, average of three individual experiments; bars, SD. a P < 0.05 vs. 0.2% DMSO; b P < 0.05 vs. 30 ng/mL TRAIL or 1.0 μmol/L AFMC alone. Figure 5B: Cells were treated with the indicated concentrations of AFMC for 24 h, DR5 and DR4 protein levels on the surface of A549 cells were determined using flow cytometry. Mouse IgG1 was used as an isotype control.

We investigated whether AFMC in combination with TRAIL induces apoptosis and increases DR5 protein levels on the surface of other NSCLC cells, including H460 and H157. Combined use of AFMC with TRAIL induced the accumulation of sub-G1 population in a dose-dependent manner in both cell lines (Figure 5A). The results by FCM analysis shown that AFMC increased DR5 protein levels dose-dependently on the surface of H460 and H157 cells, but DR4 expression levels did not obviously change (Figure 5B). Together, these findings suggest that the potentiation of TRAIL-induced apoptosis and upregulation of DR5 expression by AFMC is not specific to A549 cell line.

**Figure 6:**
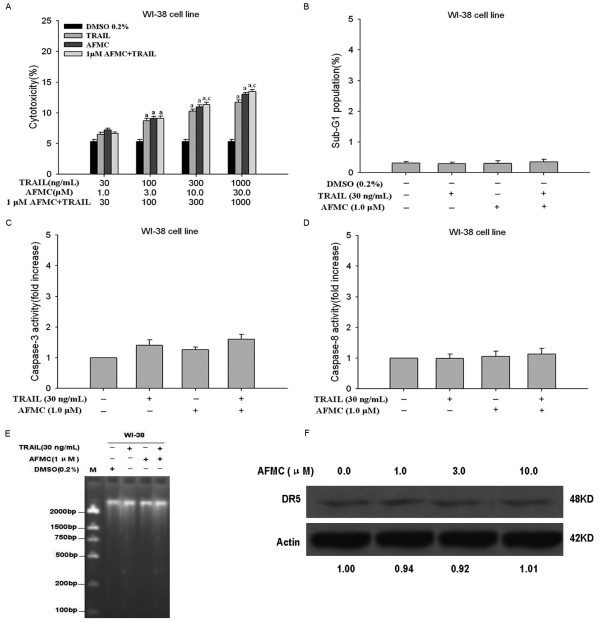
**AFMC neither induce expression of DR5 nor enhance TRAIL-induced apoptosis in WI-38 cells**. Figure 6A: WI-38 cells were treated with AFMC for 30 min and then treated with TRAIL for 24 h at the indicated concentrations. Cytotoxicity was assessed by ELISA. Columns, average of three individual experiments; bars, SD. a P < 0.05 vs. 0.2% DMSO; c P < 0.05 vs. 1.0 μmol/L AFMC. Figure 6B: WI-38 cells were treated with 1.0 μmol/L AFMC, 30 ng/mL TRAIL or the pretreated with 1.0 μmol/L AFMC for 30 min followed by 30 ng/mL TRAIL for 24 h. DNA content of the cells was analyzed by flow cytometry. Figure 6C: WI-38 cells were treated as described above. The enzymatic activity of caspase-3 was determined by incubation of 20 μg total protein with 200 μM of Ac-DEVD-pNA in 100 μL of assay buffer for 2 h at 37°C. pNA release was monitored spectrophotometrically at 405 nm. Figure 6D: WI-38 cells were treated as described above. The enzymatic activity of caspase-8 was determined by incubation of 20 μg of total protein with 200 μM of Ac-IETD-pNA in 100 μL of assay buffer for 2 h at 37°C. pNA release was monitored spectrophotometrically at 405 nm. Data are shown as mean ± S.D. (n = 3). Figure 6E: WI-38 cells were treated as described above. Fragmented DNA was extracted from the treated cells and analyzed on a 2.0% agarose gel. Figure 6F: WI-38 cells were treated with the indicated concentrations of AFMC for 24 h, and DR5 levels were assessed by Western blotting. β-actin was used as a loading control.

To evaluate the selectivity of apoptosis induction of NSCLC cells by combination treatment with AFMC and TRAIL, we next examined the cytotoxic effects of AFMC and TRAIL in human immortalized embryo lung WI-38 cells. Notably, neither TRAIL or AFMC alone nor in combination was cytotoxic to WI-38 cells (Figure 6A). In addition, the increased frequency of cells in the sub-G1 phase, activation of caspase-3 and -8 and DNA fragmentation induced by co-treatment with TRAIL and AFMC were not observed in WI-38 cells (Figure 6B, C, D, E) under conditions in which apoptosis was markedly induced in A549 cells (Figure 1C, D, E). In addition, AFMC did not induce the expression of DR5 protein in WI-38 cells (Figure 6F).

## Discussion

Flavonoids are a broad class of plant polyphenols that have important pharmacological effects. Recent studies have shown that chrysin and its derivatives possess strong anticancer activities. Methylated polyphenol derivatives have been reported to possess stronger bioactivities and higher hepatic metabolic stability and intestinal absorption than the lead compound [40,41], making these derivatives more favorable than the unmethylated lead compound for development as potential cancer chemopreventive agents. Our previous studies showed that AFMC, a derivative of chrysin, potently inhibited the proliferation of ovarian cancer CoC1 cells and human hepatocellular carcinoma HepG2 cells in a dose-dependent manner [11,42]. In this study, we found that AFMC sensitized A549 cells to TRAIL-induced apoptosis and enhanced the cytotoxic effects of TRAIL but had little effect on human embryo lung WI-38 cells (Figures 1 and 6). Combined treatment with subtoxic concentrations of AFMC and TRAIL induced caspase-3 and -8 activation and apoptosis of A549 cells (Figure 2). Activation of caspases plays an important role in apoptosis triggered by various proapoptotic signals [42,43]. Two major apoptotic pathways exist: one involving signals transduced through death receptors and one dependent on signals from the mitochondria [9,42]. Both pathways activate the caspase cascade, leading to cleavage of cellular substrates and the morphological and biochemical manifestations of apoptosis. The activation of caspase-8 and -9 has been documented to play central roles in mediating apoptosis through death receptors and the mitochondrial pathway, respectively [9,42]. However, caspase-8 can activate caspase-9-mediated apoptotic pathways via activation or cleavage of Bid protein [42,43]. Therefore, our results suggest that the induction of apoptosis in A549 cells by AFMC and TRAIL might involve death signals transduced through death receptors. As expected, we found that AFMC increased the expression of DR5 in a concentration- and time-dependent manner in A549 cells but not in WI-38 cells. In addition, we demonstrated that AFMC enhanced TRAIL-induced apoptosis in human lung cancer A549 cells through upregulation of DR5. Collectively, our findings could yield new insights into maximizing the efficiency of human lung carcinoma treatments while minimizing side effects to normal tissues.

Lung cancer represents the leading cause of cancer deaths in the world [1,2]. NSCLC accounts for approximately 75% of lung cancers [44]. About 50% of NSCLC patients are initially diagnosed with advanced or metastatic disease, and the treatment of choice is palliative chemotherapy. Apoptosis induction is one of the major objectives of modern cancer therapy, for which an important goal is to specifically induce apoptosis in malignant tumor cells but not in normal cells. For this reason, TRAIL has recently received much attention because it preferentially kills tumor cells while leaving normal cells unaffected. However, a considerable number of tumors are resistant to TRAIL-induced apoptosis [17,21]. Various conventional chemotherapeutic agents have been demonstrated to synergistically augment TRAIL-induced apoptosis through induction of DR5 expression. In this regard, AFMC is an attractive agent for inducing DR5 expression and sensitizing TRAIL-resistant A549 cells to TRAIL.

Our study has important implications in cancer treatment. As mentioned above, AFMC enables to overcome the resistance to TRAIL in NSCLC. Furthermore, more than half of all malignant tumors possess an inactivating mutation in the *p53 *gene and *p53 *modulates the sensitivity to conventional anticancer agents [45,46]. Although H157 cells harbored inactivated *p53* [32], combined treatment with AFMC and TRAIL is also useful for *p53*-deficient tumor cells.

Activation of death receptor pathway is a new strategy for targeted therapy of cancer. Our previous study demonstrate AFMC induced apoptosis of human hepatocellular carcinoma cells via inhibition of NF-κB. Studies by Srivastava et al. shown that MS-275, a histone deacetylase (HDAC) inhibitors, could sensitize TRAIL-resistant breast cancer xenografts through the involvement of both cell-extrinsic and cell-intrinsic pathways of apoptosis[47] and resveratrol regulates the expression of FOXO's target genes such as Bim, TRAIL, DR4, DR5, cyclin D1 and p27/KIP1 [48,49]. However, how AFMC induces DR5 upregulation and enhances TRAIL-induced apoptosis in NSCLC cells need to be further investigated.

Clinically, resistance to apoptosis is a major obstacle in chemotherapeutic treatments of cancers. The ability of AFMC to sensitize A549 cells to TRAIL-induced apoptosis at subtoxic concentrations makes AFMC a potentially effective preventative and/or therapeutic agent against NSCLC. However, the potential clinical implications of our studies will depend on whether or not AFMC can be safely administered to patients in high enough doses to be pharmacologically active. Oral flavonoids cannot be utilized in the clinic because of poor bioavailability because of rapid metabolism in the liver and intestinal wall. However, Walle *et al*[41]. reported that dimethoxyflavone (DMF) was clearly detected in plasma with a peak concentration of 2.5 ± 0.8 μM (mean ± SEM) at 1 h after gavage (5 mg/kg each) to rats. In that study, the area under the plasma concentration-time curve was 58.8 μg • ml^-1 ^• min. DMF was also clearly detected in the liver, lung and kidney tissues with concentrations in the liver exceeding those in the plasma by as much as seven-fold (16.5 ± 5 μM) 1 h after administration. The liver, which showed the highest accumulation of DMF, might be a site where DMF exerts its greatest activity. Therefore, additional *in vivo *studies are needed to evaluate the applicability of AFMC as a chemopreventive and/or therapeutic agent for cancer.

## Conclusions

We have shown that AFMC induces the expression of DR5 and enhances the TRAIL-induced apoptosis in human A549 lung cancer cells. Although further research is required to dissect the underlying mechanisms and evaluate the potency of AFMC and TRAIL co-treatment in a nude mouse model, collectively, these results raise the possibility that combined treatment with TRAIL and AFMC could be a promising candidate therapeutic for human lung cancer.

## Competing interests

The authors declare that they have no competing interests.

## Authors' contributions

ZHX, MFQ and FL performed the majority of experiments. JGC participated in its design and coordination and was also involved in revising the manuscript. JSZ conceived of the study and wrote the manuscript. All authors read and approved the final manuscript.

## Pre-publication history

The pre-publication history for this paper can be accessed here:

http://www.biomedcentral.com/1471-2407/11/322/prepub
